# Relationship between blood cadmium levels and bone mineral density in adults: a cross-sectional study

**DOI:** 10.3389/fendo.2024.1354577

**Published:** 2024-03-21

**Authors:** Yi Lei, Meiqian Guo, Juan Xie, Xueqing Liu, Xiang Li, Hongwu Wang, Yong Xu, Donghui Zheng

**Affiliations:** ^1^ Department of Nephrology, The Affiliated Huai’an Hospital of Xuzhou Medical University and Huai’an Second People’s Hospital, Huai’an, China; ^2^ Department of Clinical Laboratory, The Affiliated Huai’an Hospital of Xuzhou Medical University and Huai’an Second People’s Hospital, Huai’an, China; ^3^ Huai’an Key Laboratory of Chronic Kidney Disease, The Affiliated Huai’an Hospital of Xuzhou Medical University and Huai’an Second People’s Hospital, Huai’an, China

**Keywords:** blood cadmium, NHANES, bone mineral density, osteoporosis, adults

## Abstract

**Introduction:**

Osteoporosis, a disease of reduced bone mass and microstructural deterioration leading to fragility fractures, is becoming more prevalent as aging progresses, significantly increasing the socioeconomic burden. In past studies, there has been a growing awareness of the harmful effects of heavy metals on bone, with cadmium being a significant exposure factor. The purpose of this study was to look into the association between adult bone mineral density(BMD) and blood cadmium levels.

**Methods:**

Based on information from the 2013–2014, 2017–2018 NHANES, weighted multiple regression, generalized weighted modeling, and smoothed curve fitting were utilized to investigate the association between blood cadmium and femur BMD. Furthermore, subgroup analyses were conducted to investigate any differences in the associations between age, sex, race, chronic kidney disease, and diabetes.

**Results:**

In 2,146 participants, blood cadmium levels and total femur [-0.02 (-0.03, -0.01), 0.0027], femoral neck [-0.01 (-0.02, -0.00), 0.0240], femoral trochanter [-0.01 (-0.02, -0.00), 0.0042], and intertrochanteric femoral trochanter [-0.02 (-0.03, -0.00), 0.0101] BMD were negatively correlated. Subgroup analyses showed that this association was more pronounced in women, non-Hispanic white people and other Hispanics, and those with chronic kidney disease and diabetes. Our results pointed to a negative relationship between femoral BMD and blood cadmium. This negative association varied by age, sex, race, diabetes, and chronic kidney disease. In particular, bone mineral density was more significantly negatively affected by blood cadmium levels in groups with diabetes and chronic kidney disease.

**Conclusion:**

Our findings demonstrated a significant negative association between blood cadmium levels and bone mineral density in a population of U.S. adults.

## 1 Introduction

Bones are an essential part of the human body. The bones in the human body are used for hematopoiesis, protection, support, storage, and mobility. A balance between bone resorption and production preserves bone mass. Numerous variables, including nutrition, exercise, and lifestyle choices, might have an impact on it (1, 2). Progressive loss of bone mass and deterioration of the microarchitecture of the bone may accompany osteoporosis, which makes people more susceptible to fractures and incapacitation (3). With the progress of population aging, the prevalence of osteoporosis is gradually increasing, and the effects of diet and environment are gradually being emphasized.

Cadmium exploitation is a serious global environmental problem that can lead to heavy health and socio-economic burdens. Cadmium is commonly found in household waste, cadmium-containing substances emitted by industry, and even in soil and water (4). The body may absorb cadmium by means of food, smoking, occupational exposure, and other sources (5). The effects of diet and smoking on cadmium levels are significant, food leafy and root vegetables, cereals and offal are important sources of cadmium, long-term consumption of cereals and root vegetables will increase the accumulation of cadmium in the body (6). In addition, smoking is an important route of cadmium intake, the lungs have a higher cadmium absorption rate than the digestive tract, and cadmium levels in smokers are significantly higher than in non-smokers (7), and blood cadmium levels in non-smokers are less than 1 μg/L in most countries, whereas heavy smokers may have blood cadmium levels up to 7-fold higher. There are also differences in blood cadmium levels between different countries, with the average blood cadmium level of adults in the United States being lower than the average blood cadmium level of adults in Italy, Germany, Canada, and Australia (7). Numerous prior investigations have shown an association between exposure to environmental cadmium and a number of illnesses, including osteoporosis, diabetes (8, 9), kidney damage (10), and improper child growth and development (11). Among these, by damaging the renal tubular system, interfering with the metabolism of vitamin D and parathyroid hormone (PTH), and reducing intestinal calcium absorption, cadmium may directly harm bones, thus disrupting bone mineralization (12, 13). Due to this, we postulated that adult BMD is inversely correlated with blood cadmium levels.

Using the National Health and Nutrition Examination Survey (NHANES) 2013-2014 2017-2018 databases, we examined the association between blood cadmium levels and BMD in a representative sample of American individuals≥18 years of age. We also investigated whether this relationship differed by age, sex, race, diabetes, and chronic kidney disease.

## 2 Materials and methods

### 2.1 Population and study design

**Figure 1:**
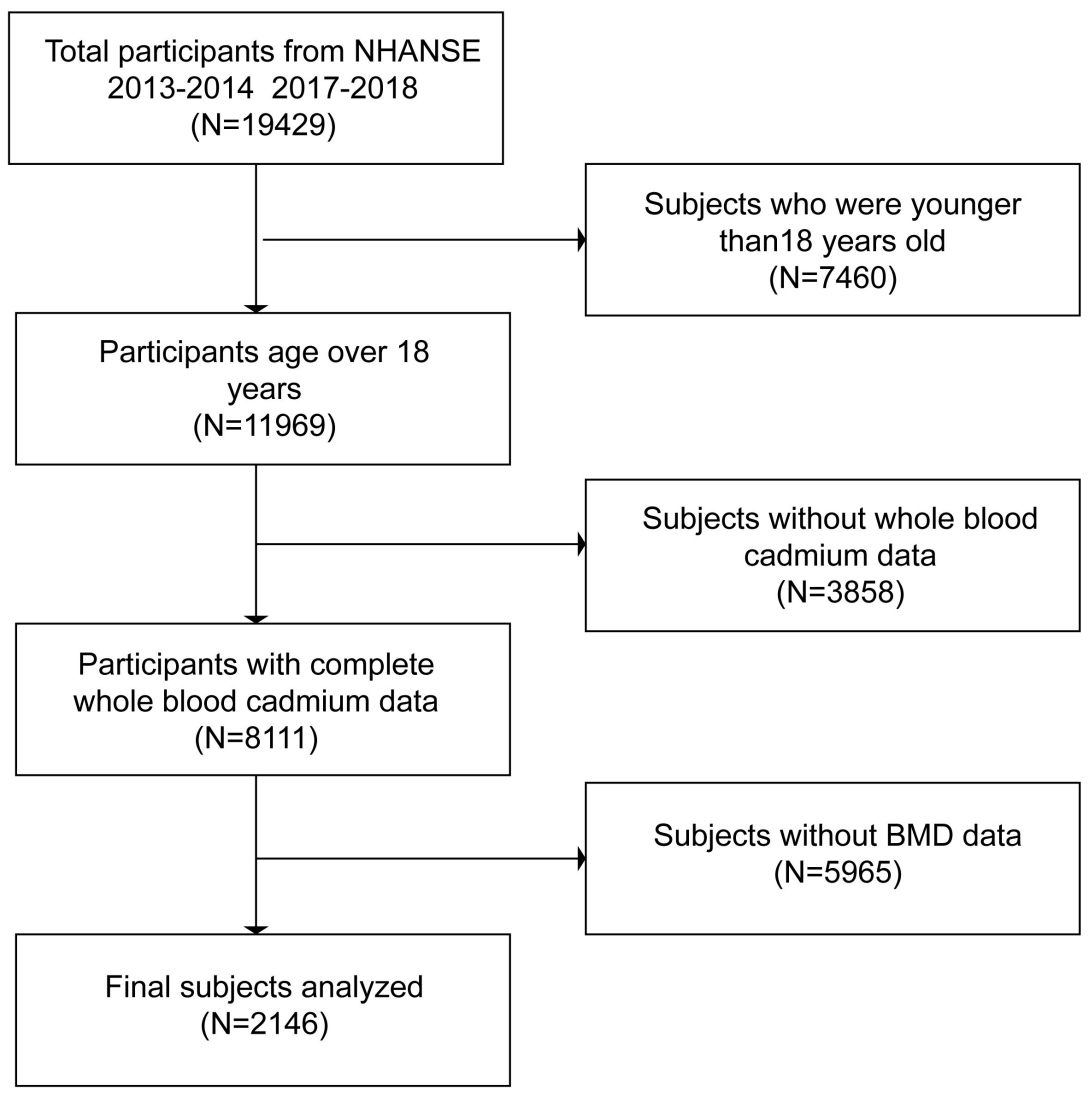
Flow chart of participants selection.

A study program called the National Health and Nutrition Examination Survey (NHANES) was created to evaluate the health and nutritional conditions of adults and children in the United States. It was carried out once a year on 5,000 or more members of a nationally representative sample. NHANES data from 2013–2014 and 2017–2018 were utilized in this investigation. Of the 19,429 participants, we excluded subjects younger than 18. A total of 8111 participants with blood cadmium data were chosen from among all subjects who were 18 years of age or older. Following that, 5,964 participants were eliminated since they lacked data on their BMD. In the end, 2,146 participants in all were recruited. All NHANES procedures were approved by the National Centre for Health Statistics Ethics Review Board, and each participant provided written informed permission (14). The selection procedure was shown in the flowchart (**Figure 1**)—relevant guidelines and regulations performed in all methods.

### 2.2 Research variables

The primary measures of cadmium exposure include blood and urinary cadmium, with urinary cadmium primarily reflecting cadmium body burdens, whereas blood cadmium changes more rapidly during cadmium exposure (7). Although urinary cadmium is considered to be a good biomarker of long-term renal accumulation, it may be influenced by several factors related to renal cadmium accumulation, such as the proportion of cadmium transferred to the kidney, the subsequent half-life in the kidney, or methods used to compensate for changes in urinary dilution, such as creatinine excretion or urine density, all of which influence changes in urinary cadmium concentrations (15). Due to the uncertainty associated with the above factors, blood cadmium is a superior biomarker of low cadmium exposure to urine cadmium when 24-hour urine is not available (16). Therefore, blood cadmium was mainly used as a cadmium exposure measure in this study. Regarding the measurement method of blood cadmium, NHANES mainly used Atomic Absorption Spectroscopy (AAS). About 5 ml of fasting venous blood was collected for heparinized anticoagulation, the supernatant was removed after centrifugation, and the cadmium ions in the plasma were converted to cadmium nitrate using nitric acid-hydrogen peroxide digestion. The processed plasma samples and the standard solution were poured sequentially into a water-cooled graphite furnace of the AAS instrument, and the absorbance was measured. The concentration of cadmium in the sample was then calculated from the standard curve. Femoral bone density was assessed by dual-energy X-ray bone densitometry (DXA). The total femur, femoral neck, femoral trochanter, and intertrochanteric femoral trochanter were the areas that were investigated. In addition, we included covariates like age, race, sex, household income poverty rate (PIR), education level, body mass index (BMI), marital status, smoking status, blood uric acid, drinking status, serum urea nitrogen, alanine aminotransferase (ALT), blood creatinine, aspartate aminotransferase (AST), urinary albumin-creatinine ratio (ACR), estimated glomerular filtration rate (eGFR. ml/min/1.73m^2^, calculated according to the Chronic Kidney Disease Epidemiology Collaboration (CKD-EPI76Cr) formula. Health status differences were also mentioned for hypertension, chronic kidney disease, and diabetes. Chronic Kidney Disease (CKD) was diagnosed based on eGFR<60 ml/min/1.73m^2^ and ACR>30 mg/g. A questionnaire was used to identify whether diabetes and hypertension were present or not. The official NHANES website provided information on interpreting, measuring, and calculating each variable (https://www.cdc.gov/nchs/nhanes/).

### 2.3 Statistical analysis

For continuous variables, the data were presented as mean ± standard deviation (SD), and for categorical variables, as numbers (percentages). Chi-square and t-tests were used to evaluate the gender-specific demographic characteristics of the individuals. Three different models of weighted multiple linear regression were employed to examine the association between blood cadmium and BMD. Model 1 did not require variable adjustment. Model II incorporated the three most common demographic variables used in clinical studies-age, gender, and race. Model III, in addition to incorporating the above three common demographic variables, also referenced some other common covariates incorporated in studies on blood cadmium and BMD correlation, such as education level, marital status, PIR, ALT, AST, BMI, BUN, uric acid, ACR, and alcohol consumption. In addition, we also included variables that were suggested to be associated with BMD in some of the previous studies, such as smoking status: a study concluded that current smokers exhibited a faster decline in BMD over 3 years compared to previous smokers (17). Diabetes status: a population-based study of East Asian men found that the mean BMD of the total hip and femoral neck in diabetic patients with a long duration of disease was significantly lower than that in diabetic patients with a short duration of disease, suggesting that the duration of diabetes was significantly correlated with reduced BMD (18). CKD status: Advanced CKD patients were highly susceptible to the adverse complications of osteoporosis (19). Subsequently, we also thought of blood cadmium as quartiles to further evaluate this relationship’s robustness. Smooth curve fitting was used to estimate the nonlinear association of blood cadmium with BMD. In addition, we performed subgroup analyses to determine the association between blood cadmium and BMD across age, sex, ethnicity, diabetes status, and CKD status groups. Empower Stats statistical software and R version 4.2.3 were used in this experiment. Statistics were deemed significant if p<0.05.

## 3 Results

### 3.1 Baseline characteristics

This study comprised a total of 2,146 individuals with complete information. **Table 1** displayed the baseline characteristics of the subjects that were included in the final analysis. The participants’ weighted mean age was 58.02 ± 10.47 years, of whom 53.22% (n=1,142) were females. The following variables showed statistically significant differences between males and females: marital status, household income to poverty ratio, alcohol consumption, smoking status, diabetes mellitus, chronic kidney disease, body mass index, AST, ALT, blood urea nitrogen, blood uric acid, blood creatinine, glomerular filtration rate, and femur BMD (all P < 0.05). Females had a higher prevalence rate of chronic kidney disease and hypertension were more educated, and had higher blood cadmium levels than males. Conversely, males were more likely to be smokers, drinkers, and to have diabetes. They also had greater levels of bone density in the total femur, femoral neck, femoral trochanter, and intertrochanteric femoral trochanter.

**Table 1:** Characteristics of the participants.

Characteristics	Male	Female	*P-value*
	(N = 1,004)	(N = 1,142)	
Age (years)	57.12 ± 10.21	58.78 ± 10.62	0.0002
Race (%)			0.1916
Mexican American	8.41	6.45	
Other Hispanic	5.51	7.19	
Non-Hispanic White	66.15	66.47	
Non-Hispanic Black	10.01	10.94	
Other Race	9.92	8.95	
Education (%)			0.0507
Less than high school	5.18	4.67	
High school or GED	36.30	31.67	
Above high school	58.52	63.66	
Marital status(%)			<0.0001
YES	70.02	57.86	
NO	29.98	42.14	
Smoked≥ 100 cigarettes in life (%)			<0.0001
YES	79.76	68.02	
NO	20.24	31.98	
Drink status(%)			<0.0001
YES	79.76	68.02	
NO	20.24	31.98	
Hypertension(%)			0.6300
YES	41.13	42.16	
NO	58.87	57.84	
Diabetes (%)			<0.0001
YES	22.29	13.47	
NO	77.71	86.53	
CKD (%)			0.0018
YES	17.85	23.35	
NO	82.15	76.65	
Income to poverty ratio	3.29 ± 1.61	3.15 ± 1.63	0.0593
Body mass index (kg/m^2^)	28.85 ± 5.33	28.34 ± 6.38	0.0456
AST(U/L)	25.74 ± 12.78	22.27 ± 9.79	<0.0001
ALT(U/L)	28.12 ± 17.62	20.38 ± 13.19	<0.0001
BUN(mg/dl)	15.39 ± 4.89	14.39 ± 5.18	<0.0001
Creatinine (mg/dl)	1.01 ± 0.36	0.80 ± 0.26	<0.0001
Uric acid (mg/dl)	15.39 ± 4.89	14.39 ± 5.18	<0.0001
ACR (mg/g)	42.41 ± 262.06	36.75 ± 326.79	0.6626
eGFR (mL/min/1.73 m^2^)	82.36 ± 19.48	80.33 ± 19.63	0.0178
Blood cadmium(ug/L)	0.43 ± 0.51	0.51 ± 0.55	0.0004
Total femur BMD (g/cm^2^ )	0.99 ± 0.13	0.87 ± 0.14	<0.0001
Femur neck BMD (g/cm^2^)	0.801 ± 0.128	0.728 ± 0.136	<0.00001
Trochanter BMD (g/cm^2^)	0.743 ± 0.114	0.659 ± 0.115	<0.00001
Intertrochanter BMD (g/cm^2^)	1.183 ± 0.157	1.041 ± 0.174	<0.00001

Mean ± SD for continuous variables: the p-value was calculated by the weighted linear regression model. (%) for categorical variables: the p-value was calculated by the weighted chi-square test.

GED, general educational development; CKD, chronic kidney disease;AST, aspartate transaminase; ALT, alanine transaminase;BUN, blood urea nitrogen; ACR, albumin: creatinine ratio; eGFR,estimated-glomerular filtration rate.

### 3.2 Relationship between blood cadmium levels and bone mineral density

**Table 2** displayed the outcomes of the multiple linear regression analysis between blood cadmium levels and BMD (total femur, femoral neck, femoral trochanter, and intertrochanter). In model 1 (unadjusted), the total femur[-0.03 (-0.05,-0.02)]; femoral neck [-0.02 (-0.03,-0.01)]; femoral trochanter[-0.03 (-0.04,-0.02)]; and intertrochanteric femur[-0.04 (-0.05,-0.02)] BMD decreased with increasing blood cadmium levels. It was shown that for every 1ug/L increase in blood cadmium, total femoral BMD decreased by 0.03g/cm^2^. This effect value was 0.02, 0.03, 0.04 for femoral neck BMD, femoral trochanter BMD, and intertrochanteric BMD, respectively. Moreover, after converting blood cadmium levels into categorical variables [quartiles, Q1 (0.07 - 0.2 μg/L), Q2 (0.21 - 0.34 μg/L), Q3 (0.35 - 0.6 μg/L), Q4 (0.61 - 7.5 μg/L)], the trend of blood cadmium in femur BMD remained significant (P<0.05 for trend). In model 2 (adjusted for age, sex, and race/ethnicity), the results showed that for every 1ug/L increase in blood cadmium, total femur BMD decreased by 0.03 g/cm^2^. This effect value was 0.02, 0.02, 0.03 for femoral neck BMD, femoral trochanter BMD and intertrochanteric BMD, respectively. This trend persisted after converting blood cadmium levels to categorical variables (P<0.05). After simultaneous adjustment for all covariates (model 3), total femur femoral neck trochanter and intertrochanter BMD still declined with increasing blood cadmium levels. However, in model 3, after the blood cadmium level was converted into a categorical variable, there was no significant trend of blood cadmium change in BMD of the total femur, femoral trochanteric, and intertrochanteric femur (P > 0.05).

**Table 2:** Association between blood cadmium (ug/L) and femur bone mineral density (g/cm^2^).

		Model 1 β (95% CI) P value	Model 2 β (95% CI) P value	Model 3 β (95% CI) P value
**Total femur BMD** **(g/cm^2^)**	**Blood cadmium (ug/L)**	-0.03(-0.05,-0.02) <0.0001	-0.03 (-0.04, -0.02) <0.0001	-0.02 (-0.03, -0.01) 0.0027
	**Q1**	Reference	Reference	Reference
	**Q2**	-0.02 (-0.03, 0.00) 0.0623	0.02 (0.01, 0.04) 0.0012	0.04 (0.02, 0.05) <0.0001
	**Q3**	-0.06 (-0.07, -0.04) <0.0001	0.01 (-0.01, 0.02) 0.3519	0.03 (0.01, 0.04) 0.0008
	**Q4**	-0.06 (-0.08, -0.05) <0.0001	-0.03 (-0.05, -0.01) 0.0002	-0.00 (-0.02, 0.02) 0.8712
	***P for trend***	<0.001	<0.001	0.858
**Femur neck BMD** **(g/cm^2^)**	**Blood cadmium** **(ug/L)**	-0.02 (-0.03, -0.01) 0.0001	-0.02 (-0.03, -0.01) 0.0002	-0.01 (-0.02, -0.00) 0.0240
	**Q1**	Reference	Reference	Reference
	**Q2**	-0.01 (-0.03, 0.00) 0.0536	0.02 (0.00, 0.03) 0.0081	0.03 (0.01, 0.04) 0.0001
	**Q3**	-0.04 (-0.06, -0.02) <0.0001	0.01 (-0.01, 0.02) 0.2934	0.02 (0.00, 0.04) 0.0167
	**Q4**	-0.04 (-0.06, -0.03) <0.0001	-0.02 (-0.04, -0.01) 0.0082	-0.00 (-0.02, 0.02) 0.8378
	***P for trend***	<0.001	0.05	0.162
**Trochanter BMD** **(g/cm^2^)**	**Blood cadmium (ug/L)**	-0.03 (-0.04, -0.02) <0.0001	-0.02 (-0.03, -0.01) <0.0001	-0.01 (-0.02, -0.00) 0.0042
	**Q1**	Reference	Reference	Reference
	**Q2**	-0.01 (-0.02, 0.00) 0.1751	0.02 (0.01, 0.03) 0.0024	0.03 (0.02, 0.04) <0.0001
	**Q3**	-0.04 (-0.05, -0.02) <0.0001	0.01 (-0.01, 0.02) 0.2789	0.03 (0.01, 0.04) 0.0001
	**Q4**	-0.05 (-0.07, -0.04) <0.0001	-0.03 (-0.04, -0.01) <0.0001	-0.00 (-0.02, 0.01) 0.9052
	***P for trend***	<0.001	<0.001	0.920
**Intertrochanter BMD** **(g/cm^2^)**	**Blood cadmium (ug/L)**	-0.04 (-0.05, -0.02) <0.0001	-0.03 (-0.04, -0.02)<0.0001	-0.02 (-0.03, -0.00) 0.0101
	**Q1**	Reference	Reference	Reference
	**Q2**	-0.02 (-0.04, 0.00) 0.0802	0.03 (0.01, 0.05) 0.0014	0.04 (0.03, 0.06) <0.0001
	**Q3**	-0.06 (-0.08, -0.04) <0.0001	0.01 (-0.01, 0.03) 0.2912	0.03 (0.01, 0.05) 0.0014
	**Q4**	-0.07 (-0.10, -0.05) <0.0001	-0.03 (-0.05, -0.01) 0.0006	-0.00 (-0.02, 0.02) 0.9907
	***P for trend***	<0.001	<0.001	0.963

Model 1: no covariates were adjusted.

Model 2: age, sex, and race were adjusted.

Model 3: age, sex, race, education, marital status, smoking status, drink status, hypertension, diabetes, CKD, Income to poverty ratio, Body mass index, ALT, AST, BUN, creatinine, uric acid, eGFR, ACR were adjusted.

CKD, chronic kidney disease; ALT, alanine aminotransferase; AST, aspartate aminotransferase; BUN, blood urea nitrogen; eGFR, estimated-glomerular filtration rate; ACR, albumin:creatinine ratio.

**Figure 2:**
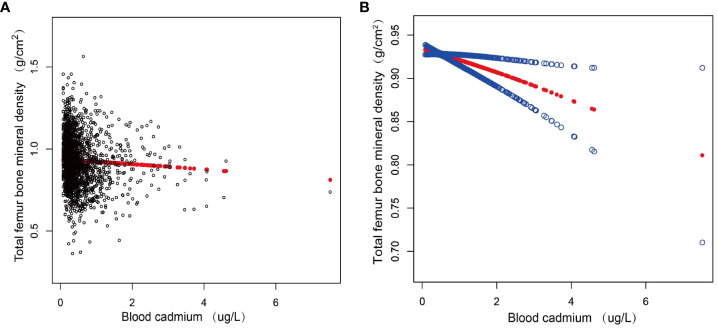
The association between blood cadmium and total femur BMD. **(A)** Each black point represents a sample. **(B)** Red line represents the smooth curve fit between variables. Blue lines represent the 95% of confidence interval from the fit. Age, sex, race, education, marital status, smoking status, drink status, hypertension, diabetes, CKD, Income to poverty ratio, Body mass index, ALT, AST, BUN, creatinine, uric acid, eGFR, ACR were adjusted. CKD, chronic kidney disease; ALT, alanine aminotransferase; AST, aspartate aminotransferase; BUN, blood urea nitrogen; eGFR, estimated-glomerular filtration rate; ACR, albumin:creatinine ratio.

**Figure 2** displayed the nonlinear association between blood cadmium and total femur BMD after correcting for all variables.

### 3.3 Subgroup analysis

As shown in **Table 3**, blood cadmium levels in both males [-0.02 (-0.04, -0.00), P<0.05] and females [-0.03 (-0.04, -0.02), P<0.05] showed a decline, with a more robust correlation observed in females compared to males. Regarding race/ethnicity, fully adjusted models for non-Hispanic white individuals and other Hispanics revealed a strong negative correlation between blood cadmium levels and total femur BMD. However, no such correlation was found in non-Hispanic black individuals and other races (including multiracial), whereas a positive association was demonstrated in Mexican Americans. When stratified by the presence of diabetes, a relevant association was also observed between blood cadmium levels and total femur BMD in populations with [-0.03 (-0.05, -0.00), P<0.05] and without diabetes [-0.01 (-0.02, -0.00), P<0.05], with a more pronounced relevance in those with diabetes. Stratified by CKD status, blood cadmium levels negatively correlated with total femur BMD in both those with CKD [-0.03 (-0.05, -0.00), P<0.05] and those without [-0.01 (-0.03, -0.00), P<0.05], with this association being more prominent in those with CKD.

**Table 3:** Association between blood cadmium (ug/L) and total femur bone mineral density (g/cm^2^) stratifed by age, sex, race, diabetes and CKD.

	Model 1 β(95%CI)P value	Model 2 β(95%CI)P value	Model 3 β(95%CI)P value
Stratified by age			
<60 years	-0.03 (-0.05, -0.02) <0.0001	-0.03 (-0.04, -0.02) <0.0001	-0.02 (-0.03, -0.00) 0.0295
≥60 years	-0.03 (-0.05, -0.01) 0.0010	-0.02 (-0.04, -0.00) 0.0136	-0.02 (-0.03, -0.00) 0.0361
Stratified by sex			
Males	-0.02 (-0.04, -0.01) 0.0040	-0.03 (-0.04, -0.01) 0.0011	-0.02 (-0.04, -0.00) 0.0294
Females	-0.03 (-0.04, -0.01) 0.0007	-0.02 (-0.04, -0.01) 0.0004	-0.03 (-0.04, -0.02) <0.0001
Stratified by race/ethnicity			
Mexican American	-0.06 (-0.12, 0.01) 0.0792	0.00 (-0.05, 0.06) 0.9279	0.08 (0.02, 0.14) 0.0162
Other Hispanic	-0.08 (-0.13, -0.02) 0.0046	-0.07 (-0.12, -0.02) 0.0033	-0.06 (-0.11, -0.02) 0.0048
Non- Hispanic White	-0.03 (-0.05, -0.01) 0.0023	-0.02 (-0.04, -0.01) 0.0067	-0.02 (-0.03, -0.00) 0.0412
Non- Hispanic Black	-0.03 (-0.05, -0.00) 0.0232	-0.03 (-0.05, -0.01) 0.0054	-0.00 (-0.03, 0.03) 0.9246
Other race/ ethnicity	-0.06 (-0.08, -0.03) <0.0001	-0.03 (-0.05, -0.01) 0.0049	-0.01 (-0.03, 0.01) 0.3728
Stratified by Diabetes			
YES	-0.04 (-0.07, -0.02) 0.0008	-0.03 (-0.05, -0.01) 0.0055	-0.03 (-0.05, -0.00) 0.0212
NO	-0.03 (-0.04, -0.02) <0.0001	-0.02 (-0.03, -0.01) 0.0001	-0.01 (-0.02, -0.00) 0.0471
Stratified by CKD			
YES	-0.04 (-0.07, -0.01) 0.0129	-0.04 (-0.07, -0.02) 0.0013	-0.03 (-0.06, -0.00) 0.0404
NO	-0.03 (-0.05, -0.02) <0.0001	-0.02 (-0.03, -0.01) <0.0001	-0.01 (-0.03, -0.00) 0.0123

Model 1: no covariates were adjusted.

Model 2: age, sex, and race were adjusted.

Model 3: age, sex, race, education, marital status, smoking status, drink status, hypertension, diabetes, CKD, Income to poverty ratio, Body mass index, ALT, AST, BUN, creatinine, uric acid, eGFR, ACR were adjusted.

CKD, chronic kidney disease; ALT, alanine aminotransferase; AST, aspartate aminotransferase; BUN, blood urea nitrogen; eGFR, estimated-glomerular filtration rate; ACR, albumin:creatinine ratio.

## 4 Discussion

We investigated the association between blood cadmium and BMD in adult US population in this nationally representative investigation. We found that blood cadmium was inversely related to BMD in the total femur, femoral neck, femoral trochanter, and intertrochanter. Reduced bone density is a risk factor for fracture occurrence, and in general, each unit of change in bone density corresponds to a corresponding increase in fracture risk. A study on a non-smoking population in Malmöå, Sweden, found that when blood cadmium was used as a continuous variable to assess the association with non-vertebral osteoporosis-related fractures, the OR corresponding to fracture risk for each 1 ug/L increase in blood cadmium was approximately 1.5-1.6 and was significant in all models (20). Thus, our findings suggested that cadmium exposure significantly increased the risk of fracture. Furthermore, subgroup studies revealed that this negative association was present in both men and women in the age ranges of under 60 and over 60, regardless of whether they had diabetes or chronic kidney disease. It’s interesting to point out that while non-Hispanic black people and other races (including multiracial) did not show this negative association, other Hispanic and non-Hispanic white people did. In contrast, a positive association was demonstrated in Mexican Americans. In addition, higher blood cadmium levels in females with diabetes or chronic renal disease increased their risk of osteoporosis and total femur bone loss.

Several previous studies have found a negative correlation between blood cadmium levels and bone mineral density demonstrated in different populations. Dietary cadmium exposure was positively associated with fracture risk in a Swedish mammography cohort population (21), and Eunae found a significant negative correlation between blood cadmium and bone mineral density in a group of young and middle-aged men (22), and that cadmium exposure significantly increased the risk of fracture (23). An analysis of 83 male workers with occupational cadmium exposure showed that increased urinary cadmium excretion was significantly connected with a subsequent reduction in BMD and increased risk of osteoporosis (24). Another cross-sectional study conducted in the United States revealed a dose-dependent relationship between bone trabecular scores and blood cadmium in both males and females (p < 0.05 for both trends), and a substantial inverse relationship between bone trabecular scores and blood cadmium revealed this association (p < 0.05) (25). Furthermore, a follow-up study including 488 females in southeast China revealed a noteworthy correlation between urinary cadmium and osteoporosis in females (26). The results of a study in a population of postmenopausal women in Korea showed a strong association between the risk of osteoporosis and bone loss and blood cadmium levels (27). Sommar et al. found a strong association between fracture risk and erythrocyte cadmium (Ery-Cd) in a study of a prospective sample from the Health and Disease Research Biobank in northern Sweden, with subgroup analyses suggesting that cadmium is a risk factor for hip fracture in women (28); Engström et al. found that dietary cadmium exposure was negatively correlated with whole body and lumbar vertebral bone mineral density negatively and that even low levels of dietary cadmium exposure were associated with low BMD and increased risk of osteoporosis and fractures (21); Another study found that lifetime low-level exposure to cadmium reduces bone mass during skeletal growth and affects bone metabolism during maturation, leading to bone loss, and that long-term low-level cadmium exposure may be an important factor in increasing the risk of lumbar osteoporosis with vertebral deformity and fracture in the elderly (23). Ougier et al. assessed the cost of the cadmium-related burden of osteoporosis-associated fractures in three European countries (Belgium, France, and Spain) based on the Human Biomonitoring (HBM) study conducted in three countries, suggesting that cadmium exposure plays a major contributory role in the overall societal costs associated with osteoporosis (29). These findings were consistent with our own. According to our findings, there was a negative relationship between blood cadmium and BMD in both males and females, and that BMD in females was more affected by blood cadmium levels than in males. Race and sex also had an impact on the association between cadmium levels and bone loss, according to a new meta-analysis based on worldwide epidemiology research (30). Our subgroup analysis revealed significant racial disparities in the negative association between blood cadmium levels and BMD among U.S. adults, predominantly among other Hispanics and non-Hispanic white people. In addition, this negative relationship between blood cadmium and BMD varied in populations with and without diabetes or chronic kidney disease.

Currently, mechanisms exploring the association between cadmium exposure and BMD are unclear. It has been shown that cadmium can directly increase bone resorption and inhibit bone formation by promoting receptor-activated nuclear factor kappa B ligand expression (RANKL) and inhibiting osteoprotegerin (OPG) expression (31). The Wnt/b-catenin signaling pathway is essential for bone mass formation and osteoblastic differentiation and plays a key role in bone mineralization, structure and physiology, cadmium is able to impede the bone mineralization process by interfering with the Wnt/b-catenin signaling pathway thereby affecting bone formation (32). In addition, cadmium can disrupt the balance between bone formation and bone turnover by stimulating the differentiation and activity of osteoclasts and inhibiting the differentiation and activity of osteoblasts while inducing osteoblast-like cells to secrete a substance that stimulates the formation of bone-resorbing cells (33). Cadmium has also been linked to the overproduction of free radicals in bone and other tissues and to oxidative stress, which increases free radicals in osteoblasts and thus induces apoptosis and increased bone fragility (34). Other studies have shown that chronic exposure to cadmium also produces neurotoxic effects (35). The sympathetic nervous system plays an important role in bone metabolism, osteogenesis and bone reconstruction, and loss of sympathetic nerves affects vasoregulation and, consequently, blood supply to the skeleton (36). Sex differences in the correlation between blood cadmium and bone mineral density may be related to the estrogen pathway, cadmium at low micromolar concentrations has been shown to activate the estrogen receptor (ER) in cultured MCF7 breast cancer cells (37). In addition, Cd has a very high affinity for the isolated ER, and *in vitro*, Cd also blocks the binding of estrogen to its receptor, thereby interfering with the regulatory effects of estrogen on bone metabolism (38). Other theories include that cadmium causes renal dysfunction through damage to renal tubular epithelial cells, which diminishes calcium reabsorption by renal tubular cells and impedes calcium absorption from the digestive tract (39, 40). Besides, cadmium exposure interferes with parathyroid hormone (PTH), which affects the production of active vitamin D, further leading to bone loss (41). Rats treated with cadmium had an increase in malondialdehyde levels and a decrease in antioxidant enzyme activity, which in turn affected the survival and proliferation of bone marrow mesenchymal stem cells (BMMSCs), according to animal tests and *in vitro* cellular assays (42). Meanwhile, via overactivating the NF-κB signaling pathway, cadmium accelerated cellular senescence, enhanced adipogenesis of BMMSCs, and hindered osteogenic differentiation (43). However, in regard to the causes of the ethnic variations in blood cadmium levels and BMD, it has been hypothesized that such differences might be connected to the reality that cadmium exposure causes dissimilar degrees of renal tubular damage in different racial populations (44).

In our research, we also conducted a stratified analysis of the diabetes population and the chronic kidney disease population. According to our findings, people with diabetes who had higher blood cadmium levels had a greater risk of acquiring osteoporosis. People with type 1 diabetes had a higher chance of acquiring osteoporosis, according to research done in the United States on 2050 individuals with diabetes (45). Additionally, the mean BMD of the entire femoral neck and hip in diabetic patients with >5 years of disease was substantially lower than in diabetic patients with ≤5 years of illness, according to a population-based study involving 3,383 males from East Asia. This finding suggested that the length of diabetes had a strong correlation with reduced BMD (18). Based on these results, persistent diabetic status may adversely affect BMD, thereby increase the risk of osteoporosis development. The underlying pathophysiological mechanisms of decreased BMD due to diabetes are complicated and include oxidative stress, hyperglycemia, and the buildup of advanced glycosylation end-products, which lead to decreased collagen quality, increased bone marrow obesity, the release of inflammatory mediators and adipokines from visceral fat, and may be altered osteoblast function (46, 47). In addition, stratified analyses showed that changes in BMD in the chronic kidney disease population were more susceptible to cadmium levels. According to earlier research, CKD patients had a much greater incidence of osteoporosis than age-matched controls, bone density loss begins at an early stage, and osteoporosis was independently associated with CKD (48, 49). The uremic milieu, anomalies in vitamin D metabolism, imbalances in the calcium-phosphorus ratio, and parathyroid hormone are important factors that contribute to renal bone disease (50). Moreover, sex hormone disorders in patients with CKD and the use of various medications such as hormones, diuretics, vitamin K antagonists, heparin, and proton pump inhibitors may give rise to osteoporosis (51). One of the possible causes underlying the development of osteoporosis in people with chronic kidney disease is the early rise of serum fibroblast growth factor-23 levels in these patients. This inhibition of vitamin D’s activation leads to an increase in its catabolism (52).

In examining the association between blood cadmium status and BMD in various populations, this research offers a number of advantages. First, the sample for this research was drawn using multistage random sampling, and the data had a high degree of reliability and normality and were representative of the overall U.S. population. Second, this research stratified for age, sex, race, diabetes, and chronic kidney disease in order to evaluate variations in the association between blood cadmium status and BMD in various individuals after appropriately correcting for confounding variables. Furthermore, this research discovered that there was a consistent association between patients’ increased blood cadmium levels and decreased femoral BMD in a variety of groups, indicating that blood cadmium may be useful in predicting fragility fractures, bone loss, and osteoporosis. Nonetheless, it is essential to acknowledge the constraints of our research. First, a causal association between blood cadmium levels and BMD was unable to be established owing to the cross-sectional design of this investigation. Second, the findings were skewed because we neglected to account for factors including diet, activity, and calcium consumption. Further investigation into splitting the female participants into menopausal and postmenopausal phases was not conducted since we were unable to include menopause as a variable in the model because of the significant quantity of missing data. Finally, because only Americans were included in the research, findings may not fully apply to other nations or areas, such as Asia and Africa.

## 5 Conclusion

Our findings indicated a negative relationship between blood cadmium levels and femur BMD. This association differed in age, sex, race, diabetes, and CKD. In particular, BMD was more dramatically negatively affected by blood cadmium levels in the groups with diabetes and chronic kidney disease. We were unable to establish a causal association between blood cadmium and BMD owing to the cross-sectional research design. Consequently, further research is required to determine the potential consequences of blood cadmium on bone metabolism in the future.

## Data availability statement

Publicly available datasets were analyzed in this study. This data can be found here: Centers for Disease Control and Prevention (CDC), National Center for Health Statistics (NCHS), National Health and Nutrition Examination Survey (NHANES), https://wwwn.cdc.gov/nchs/nhanes/Default.aspx, NHANES 2013-2014 and NHANES 2017-2018.

## Ethics statement

The studies involving humans were approved by The National Center for Health Statistics. The studies were conducted in accordance with the local legislation and institutional requirements. The participants provided their written informed consent to participate in this study.

## Author contributions

YL: Writing – review & editing, Writing – original draft, Methodology, Formal analysis, Data curation, Conceptualization. MG: Writing – review & editing, Formal analysis, Data curation. JX: Writing – review & editing, Resources, Investigation, Conceptualization. XQL: Writing – review & editing, Methodology, Data curation. XL: Writing – review & editing, Supervision, Conceptualization. YX: Writing – review & editing, Supervision, Methodology. HW: Writing – review & editing, Methodology. DZ: Writing – review & editing, Supervision, Conceptualization.
